# Exploring crop genomes: assembly features, gene prediction accuracy, and implications for proteomics studies

**DOI:** 10.1186/s12864-024-10521-w

**Published:** 2024-06-19

**Authors:** Qussai Abbas, Mathias Wilhelm, Bernhard Kuster, Brigitte Poppenberger, Dmitrij Frishman

**Affiliations:** 1 Chair of Bioinformatics, TUM School of Life Sciences, Technical University of Munich, Freising, Germany; 2 Computational Mass Spectrometry, TUM School of Life Sciences, Technical University of Munich, Freising, Germany; 3 Munich Data Science Institute, Technical University of Munich, Garching, Germany; 4 Chair of Proteomics and Bioanalytics, TUM School of Life Sciences, Technical University of Munich, Freising, Germany; 5 Biotechnology of Horticultural Crops, TUM School of Life Sciences, Technical University of Munich, Freising, Germany

**Keywords:** Crop genomics, Genome annotation, Gene prediction, Plant evolution, Peptide identification, Bioinformatics algorithms

## Abstract

Plant genomics plays a pivotal role in enhancing global food security and sustainability by offering innovative solutions for improving crop yield, disease resistance, and stress tolerance. As the number of sequenced genomes grows and the accuracy and contiguity of genome assemblies improve, structural annotation of plant genomes continues to be a significant challenge due to their large size, polyploidy, and rich repeat content. In this paper, we present an overview of the current landscape in crop genomics research, highlighting the diversity of genomic characteristics across various crop species. We also assessed the accuracy of popular gene prediction tools in identifying genes within crop genomes and examined the factors that impact their performance. Our findings highlight the strengths and limitations of BRAKER2 and Helixer as leading structural genome annotation tools and underscore the impact of genome complexity, fragmentation, and repeat content on their performance. Furthermore, we evaluated the suitability of the predicted proteins as a reliable search space in proteomics studies using mass spectrometry data. Our results provide valuable insights for future efforts to refine and advance the field of structural genome annotation.

**Supplementary Information:**

The online version contains supplementary material available at 10.1186/s12864-024-10521-w.

## Background

Plant genomics is a rapidly evolving field with the potential to accelerate crop improvement and revolutionize agriculture [1–3]. A better understanding of the genetic basis of plant development and stress response pathways holds promise for creating novel crop cultivars with improved yield quantity and stability, including heightened resistance to diseases [4] and enhanced tolerance to abiotic stress [5].

The release of the first plant reference genome of *Arabidopsis thaliana* in December 2000 marked the beginning of the plant genomics era [6]. Over the past years, the number of sequenced and assembled plant genomes has been exponentially growing [7]. Several major projects are currently in progress, aimed at deepening our knowledge of plant genomics and enhancing taxonomic representation. The Darwin Tree of Life project aims to sequence the genomes of 70,000 eukaryotic species in Britain and Ireland [8]. The 10K Plant Genomes Project is set to sequence over 10,000 genomes from every major clade of plants and eukaryotic microbes, aiming to address key questions in plant evolution [9]. Additionally, the Open Green Genomes Initiative (OGG) plans to produce at least thirty-five high-quality genome assemblies and annotations across all major evolutionary lineages of land plants. Another significant initiative is the 3k Rice Genome Project, which aims to sequence 3,000 diverse Asian cultivated rice genomes [10]. This project provides substantial genomic data crucial for the discovery of novel alleles and advancing rice breeding technologies. Together, these projects represent significant strides in plant genomic research, contributing to a broader understanding and utilization of plant genetic resources. As of January 2024, GenBank [11] hosted 1781 sequenced plant species within the Viridiplantae clade, with some plant species having more than one genome assembly. These include agriculturally and economically important orders such as the Brassicales, Cucurbitales, Fagales, Malvales, Rosales, and Solanales. Additionally, further sequenced plant genomes have also been deposited in other databases, such as Phytozome [12] and PLAZA [13], or in species-specialized portals, such as The Sugarcane Genome Hub [14] and the CuGenDBv2 database [15], which hosts the reference genomes of members of the Cucurbitaceae family, consisting of economically important fruit and vegetable crops. In addition to quantity, the quality of assembled genomes has also seen a marked improvement despite the challenges posed by diverse characteristics of plant genomes, such as varying sizes, ploidy levels, heterozygosity, and high repeat content [16]. Recent advancements in telomere-to-telomere (T2T) genome completion have advanced the field, enabling the assembly of complete and accurate genomic sequences. For instance, a recent T2T assembly of the maize genome using ultralong reads from Oxford Nanopore Technology (ONT) and PacBio HiFi reads resulted in a complete genome assembly where each chromosome is represented as a single contig with a base accuracy over 99.99% [17]. This has unveiled the full complexity of highly repetitive regions and enabled the assembly of challenging genomic features such as the nucleolar organizer regions and centromeres, setting a new standard in genomic research. Moreover, similar T2T assemblies have been achieved in other plant species, such as sorghum and melon, where high-coverage sequencing technologies combined with sophisticated computational approaches have resulted in gap-free genomes [18, 19]. Out of the 1,781 genome assemblies in GenBank, 34 plants had complete genome-level assemblies, 772 had chromosome-level assemblies, 723 had scaffold-level assemblies, and 252 had contig-level assemblies.

Annotating plant genomes to identify functional elements, such as genes and regulatory regions, is a critical step for downstream studies, including proteomics and comparative genomics, offering insights into evolution and biological functions across different species [20]. Developing robust, standardized, and scalable methods for genome annotation across multiple genomes is critical for avoiding errors in gene boundaries and splice sites, thus ensuring that genome editing techniques, such as CRISPR, are effectively employed in crop genetic studies and molecular breeding [21, 22], as errors in annotations can propagate through databases, potentially misleading breeding strategies. Furthermore, through the development of standardized genome annotation methods, researchers can construct comprehensive pan-genomes, integrating detailed genes and transposable elements annotations. This approach facilitates a deeper exploration of genetic diversity, distinguishing core genes shared across populations from dispensable ones unique to specific individuals, thereby shedding light on the genetic bases of crucial agricultural traits. Over the past few decades, genome annotation has significantly advanced, enriched by data from transcriptomics, proteomics, and epigenomics [23]. Additionally, the adoption of deep learning techniques for tasks such as motif discovery and gene model prediction signifies a notable advancement in the field [24–26]. Despite the remarkable progress made in automated genome annotation tools, genome annotation remains prone to errors, especially with respect to gene boundaries, alternative splice sites, and non-coding elements. These limitations may stem from factors such as the quality of draft genome assemblies and the complex nature of eukaryotic exon maps, particularly those with large genomes and high GC content, such as those of many plant species. These genomes are often rich in repetitive elements, which complicates the precise identification and annotation of genes and regulatory elements. Furthermore, to achieve accurate predictions, genome annotation requires significant investments of time, resources, and manual curation to rectify errors and ambiguities. Consequently, there is a critical need to systematically evaluate and benchmark various gene prediction strategies to assess their reliability and to pinpoint the most effective approaches. Furthermore, this evaluation serves the purpose of limiting the propagation of errors within protein databases.

Here, we present a comprehensive analysis of the genomic data for 100 crop plants, going beyond the typical model species to encompass some of the more complex genomic structures. This effort is part of the “The Proteomes that Feed the World” project [27], which aims to chart the proteomes of the 100 most important crops for human nutrition. We examined the effects of various genomic properties, including assembly length, assembly quality, fragmentation, GC content, percentage of repetitive elements, and Angiospermae class (monocotyledons or dicotyledons), on the accuracy of structural genome annotation. Furthermore, we evaluated the suitability of the predicted proteins as a reliable search space in proteomics studies using mass spectrometry data.

## Methods

### Data collection

The list of the most important crops for human nutrition was obtained from the Food and Agriculture Organization (FAO) of the United Nations [28]. These crops were ranked according to their annual production in millions of tons for the year 2018, with our focus directed towards the top 100 crops in this ranking.

Publicly available genome assemblies of the 100 crops were systematically sourced from a variety of databases, primarily GenBank [11]. The most recent versions of assemblies, accompanied by relevant metadata on sequencing technology and assembly tools, were collected (Supplementary Table 1).

Based on the availability of genomic information and annotations, our list of crops was categorized into three distinct groups. Group A comprises crops for which both genome assemblies and annotations are publicly available. We refer to the best available version of annotation as the reference annotation, which is usually generated using comprehensive pipelines incorporating transcriptomics and/or proteomics data. Group B consists of crops that possess a genome assembly but lack a reference annotation. Finally, group C includes crops for which no publicly available genome assemblies could be found (Supplementary Table 1).

### Construction of the phylogenetic tree

NCBI taxonomy IDs for the 100 crops were collected and utilized to construct a phylogenetic tree using the PhyloT tree generator tool [29]. The tree was visualized and annotated using the interactive Tree Of Life (iTOL) tool [30].

### Quality assessment and genome statistics

The contiguity of the genome assemblies was assessed for plants in groups A and B using Quast [31]. Detailed information on the genomic features, including the assembly size, number of contigs/scaffolds, N50, L50, and GC content, can be found in Supplementary Table 2. Given that N50 values (expected: as high as possible, approaching the genome size) and L50 values (expected: as small as possible, close to 1) do not assess completeness or correctness of the assemblies, we employed BUSCO version 5.4.3 [32] to evaluate gene space completeness. This analysis used the embryophyte lineage (embryophyta_odb10) database, encompassing 1614 genes. Genomes with a completeness score exceeding 95% are considered of a high-quality. Although a completeness range of 90-95% is still acceptable, scores below 90% suggest significant deficiencies in the assembly. Additionally, due to the abundance of repetitive sequences within plant genomes, which presents significant challenges to the assembly process, it becomes imperative to evaluate the assembly of repeat space. The LAI score serves as a robust metric for assessing the continuity of intergenic and repetitive sequence assembly, facilitating cross-species comparisons. Assemblies are categorized based on their LAI score: those scoring below 10 are classified as draft quality, while assemblies falling within the range of 10 to 20 are considered reference quality. Exceptionally high-quality assemblies, denoted as gold quality, have LAI scores exceeding 20 [33]. Identification of Long Terminal Repeat retrotransposon (LTR-RT) candidates was conducted using LTRharvest [34] and LTR_FINDER [35] algorithms, with parameters detailed in supplementary file 1. Subsequently, filtering was employed via LTR_retriever [36] to isolate high-confidence LTR retrotransposons. The raw LTR-Associated Index (Raw LAI) and the corrected LAI were then computed by the LAI program deployed in the LTR_retriever package.

### Repeat annotation and masking

Repeat masking is a critical step in genome annotation as it reduces the number of erroneous gene models in subsequent steps. *De novo* repeat identification was conducted using RepeatModeler2 (version 2.0.3) [37] with subsequent annotation and soft masking of the identified repeats using RepeatMasker [38]. The percentage of repetitive sequences in each genome within groups A and B was calculated, and the prevalence of different repeat families is reported in Supplementary Table 3.

### Gene prediction

The accuracy of the state-of-the-art gene prediction tools was benchmarked using the *Arabidopsis thaliana* and *Medicago truncatula* genomes, which are well-known model plants with high-quality reference annotations curated by specialized consortia—AraPort (Arabidopsis Information Portal) [39] and the International Medicago Genome Annotation Group (IMGAG) [40]. Due to the limited availability of species-specific RNA sequencing data for most crops, gene prediction tools capable of performing ab initio prediction without extrinsic evidence, as well as those capable of utilizing hints from alignments against generic protein databases, were selected. This approach ensures the applicability and relevance of the selected tools to annotate our list of crops genomes, for which species-specific RNA-seq data may be scarce. The selected gene prediction tools for evaluation included BRAKER2 [41], GALBA [42, 43], SNAP [44], GeneID [45], GlimmerM [46], and Helixer [26]. Each tool was executed using the most appropriate species model, with all other parameters set to default values (see Supplementary File 1 for more details). The top-performing tools were chosen to annotate the crop genomes in group A, as these crops have publicly available reference annotations, which serve as essential benchmarks for standardized comparative analyses.

### Evaluation of gene prediction tools

The performance of the selected gene prediction tools was assessed at both the coding region (CDS) and the gene levels using the following standard statistical measures:

$$\text{S}\text{e}\text{n}\text{s}\text{i}\text{t}\text{i}\text{v}\text{i}\text{t}\text{y} \left(\text{S}\text{n}\right) = \frac{\text{T}\text{P}}{\text{T}\text{P}+\text{F}\text{N}}$$

$$\text{S}\text{p}\text{e}\text{c}\text{i}\text{f}\text{i}\text{c}\text{i}\text{t}\text{y} \left(\text{S}\text{p}\right) = \frac{\text{T}\text{P}}{\text{T}\text{P}+\text{F}\text{P}}$$

$$\text{F}1 \text{s}\text{c}\text{o}\text{r}\text{e} = \frac{2\text{*}\text{S}\text{n}\text{*}\text{S}\text{p}}{\text{S}\text{n}+\text{S}\text{p}}$$

At the CDS level, true positives (TP) were identified as coding regions with boundaries that precisely matched those specified in the reference annotation. Conversely, false positives (FP) were characterized as coding regions whose boundaries deviated from the reference annotation.

Additionally, at the CDS level, we calculated three supplementary metrics for sensitivity and specificity (Supplementary Fig. S1). The first metric, ‘OScore’, assesses the proportion of predicted exons that are not true positives but still overlapping with actual exons in the reference annotation, referred to as Overlapping Predicted Exons (OPE). The second metric ‘WScore’, evaluates the proportion of falsely predicted exons that do not overlap with any exons in the reference annotation, denoted as Wrong Exons (WE). The third metric ‘MScore’, evaluates the missed true exons that are overlooked by the tools and have no overlap with any predicted exon, referred to as Missed Exons (ME).

$$\mathbf{O}\mathbf{S}\mathbf{c}\mathbf{o}\mathbf{r}\mathbf{e}=\frac{\text{N}\text{u}\text{m}\text{b}\text{e}\text{r } \text{o}\text{f } \text{O}\text{v}\text{e}\text{r}\text{l}\text{a}\text{p}\text{p}\text{i}\text{n}\text{g } \text{P}\text{r}\text{e}\text{d}\text{i}\text{c}\text{t}\text{e}\text{d } \text{E}\text{x}\text{o}\text{n}\text{s } \left(\text{O}\text{P}\text{E}\right)}{\text{N}\text{u}\text{m}\text{b}\text{e}\text{r } \text{o}\text{f } \text{f}\text{a}\text{l}\text{s}\text{e}\text{l}\text{y } \text{P}\text{r}\text{e}\text{d}\text{i}\text{c}\text{t}\text{e}\text{d } \text{E}\text{x}\text{o}\text{n}\text{s } \left(\text{F}\text{P}\right)}$$

$$\mathbf{W}\mathbf{S}\mathbf{c}\mathbf{o}\mathbf{r}\mathbf{e}=\frac{\text{N}\text{u}\text{m}\text{b}\text{e}\text{r } \text{o}\text{f } \text{W}\text{r}\text{o}\text{n}\text{g } \text{E}\text{x}\text{o}\text{n}\text{s } \left(\text{W}\text{E}\right)}{\text{N}\text{u}\text{m}\text{b}\text{e}\text{r } \text{o}\text{f }\text{f}\text{a}\text{l}\text{s}\text{e}\text{l} \text{y }  \text{P}\text{r}\text{e}\text{d}\text{i}\text{c}\text{t}\text{e}\text{d } \text{E}\text{x}\text{o}\text{n}\text{s } \left(\text{F}\text{P}\right)}$$

$$\mathbf{M}\mathbf{S}\mathbf{c}\mathbf{o}\mathbf{r}\mathbf{e}=\frac{\text{N}\text{u}\text{m}\text{b}\text{e}\text{r } \text{o}\text{f } \text{M}\text{i}\text{s}\text{s}\text{e}\text{d } \text{E}\text{x}\text{o}\text{n}\text{s } \left(\text{M}\text{E}\right)}{\text{N}\text{u}\text{m}\text{b}\text{e}\text{r } \text{o}\text{f } \text{a}\text{l}\text{l } \text{R}\text{e}\text{f}\text{e}\text{r}\text{e}\text{n}\text{c}\text{e } \text{E}\text{x}\text{o}\text{n}\text{s }(\text{T}\text{P}+\text{F}\text{N})}$$

At the gene level, TP were defined as genes in which the boundaries of all coding regions corresponded exactly to the reference annotation. Conversely, FP were characterized as genes that had at least one coding region with boundaries deviating from the reference annotation.

Additionally, we compared the completeness of the predicted gene sets to the completeness of the reference annotations by running BUSCO in the protein mode with the embryophyte lineage.

We proceeded by conducting statistical comparisons to assess the performance of the selected gene prediction tools. Initially, we evaluated the normality distribution of the data for each metric using the Shapiro-Wilk test. Subsequently, we employed the most appropriate statistical tests.

### Assessment of gene prediction tools through peptide identification

The quality and reliability of the gene prediction tools can be further assessed by examining how well the predicted proteins serve as a reliable search space for identifying peptides in proteomics research. This evaluation involves analyzing the overlaps between peptides identified through the use of the reference or predicted protein sequences. A high degree of overlap indicates that the predicted protein sequences serve as a reliable search space for peptide identification.

Label-free tandem mass spectrometry datasets from thirty tissues of *Arabidopsis thaliana* (720 runs) [47] and six tissues of *Medicago truncatula* (60 runs) [48] were processed using MSFragger software [49], with all default settings except for precursor true tolerance (20 ppm) and fragment mass tolerance (20 ppm) for *Arabidopsis thaliana* and precursor true tolerance (20 ppm) and fragment mass tolerance (0.35 Dalton) for *Medicago truncatula*. These adjustments were tailored to the specifications of the mass analyzers employed in the experiments. Searches were performed against several FASTA databases, including extracted protein sequences from the reference annotations, predicted proteins derived from genome annotation tools, and the six-frame translation of the respective genomes. Each database comprised both canonical and isoform sequences, with an equivalent number of decoy sequences generated using the DecoyPyrat tool [50].

Initially, the False Discovery Rate (FDR) was set to 1 (equivalent to 100%) to permit subsequent rescoring of peptide-spectrum matches (PSMs) using the Oktoberfest pipeline [51]. Following this rescoring process, we filtered the identified PSMs, maintaining an FDR of 1%.

## Results

### Data overview

**Fig. 1:**
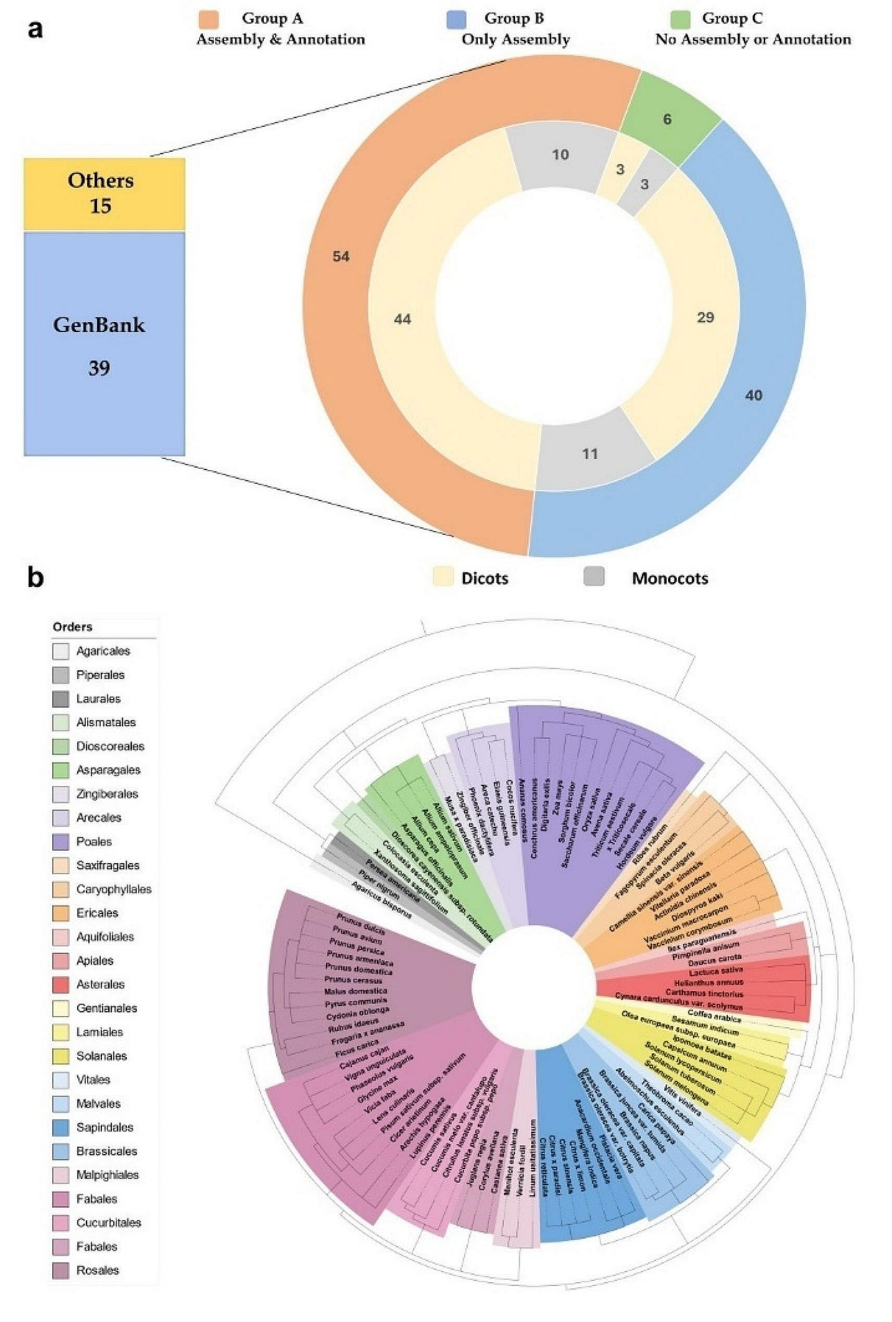
Data availability and diversity. **(a)** Categorization of 100 crops into groups based on the availability of genome assemblies and annotations, highlighting the diversity among monocotyledons and dicotyledons. **(b)** Phylogenetic spectrum of the studied crop species across diverse plant orders

Among the 100 crops studied, we were able to retrieve genome assemblies and annotation data for 54 crops, classifying them under Group A. Group B consisted of 40 crops for which genome assemblies were available but lacked annotation data. Finally, Group C included 6 crops for which neither genome assemblies nor annotations were found (Fig. 1a).

Our list of crops displays a remarkable spectrum of diversity within both the monocotyledons and dicotyledons classes (Fig. 1a; Supplementary Table 1), spanning a wide range of phylogenetically distinct orders (Fig. 1b; Supplementary Table 1). Prominent plant orders include Fabales, Poales, and Rosales. Notably, the *Agaricus bisporus* (Mushroom) cultivar stands apart from other crops due to its distant relationship with these plants, as it belongs to the Fungi kingdom.

### Sequencing technologies and assembly methods

**Fig. 2:**
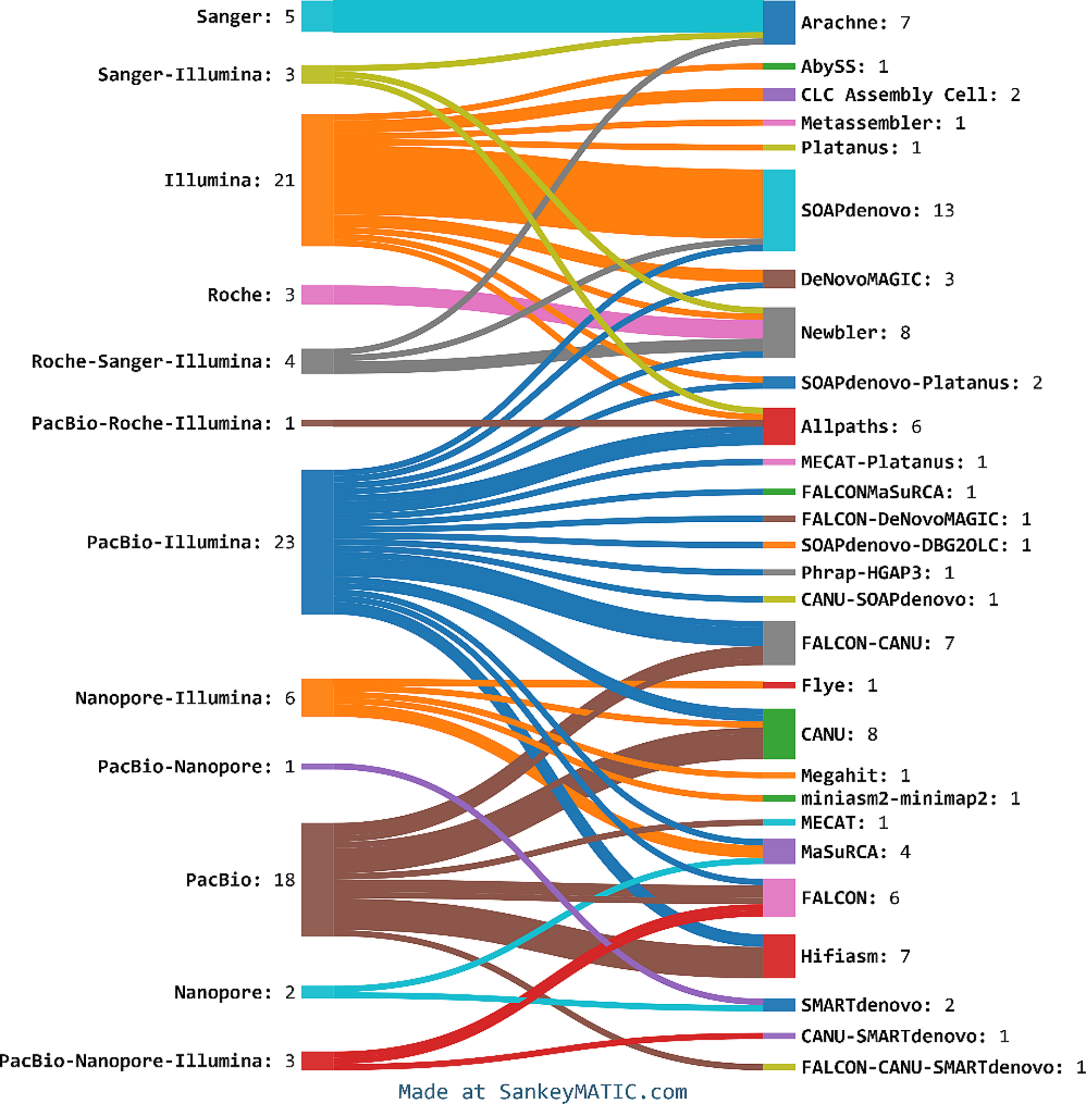
Sankey diagram illustrating the distribution of the studied genomes across various sequencing technologies from 2000 to 2024, along with the primary tools utilized for assembly

Over the past decade, crop genome sequencing strategies have undergone a significant shift, transitioning from the traditional and labor-intensive Sanger sequencing—which had been used to sequence only five genomes of our crops—to the more efficient next-generation sequencing (NGS) technologies, predominantly Illumina platforms (Fig. 2). These platforms have played a crucial role, having been utilized to sequence numerous crops, either solely (21 crops) or in combination with other methods (40 crops). With the advent of third-generation sequencing technologies, such as PacBio and Oxford Nanopore, a notable shift towards these newer methods was observed. PacBio has seen extensive use; 18 plants were completely sequenced using PacBio alone, and an additional 23 plants were sequenced using a hybrid approach combining both Illumina and PacBio technologies. In contrast, the Oxford Nanopore technology has been employed for complete sequencing in only two plants.

In terms of genome assembly methodologies, early Sanger-sequenced genomes predominantly employed the ARACHNE assembler [52], while Illumina sequencing saw extensive use of SOAPdenovo [53], Newbler [54], and Allpaths [55] as the key tools for genome assembly. However, with the advent of long-read sequencing technologies, tools like Falcon [56], Canu [57], and Hifiasm [58] have become the most commonly employed for assembling complex genomes, either independently or in combination with other tools for scaffolding and assembly polishing. Additionally, other assembly tools such as MaSuRCA [59] and SMARTdenovo [60], while used less frequently, have also gained popularity in the genomics community. The integration of these diverse tools has been critical in addressing challenges associated with complex genome structures and in enhancing the contiguity and accuracy of the assembled genomes. Moreover, recent genome assemblies almost universally employ Hi-C data to correct assembly errors and to complement linked reads and optical maps, significantly improving the scaffolding of contigs and providing chromosome-spanning contiguity to the assemblies.

### Genome statistics and quality assessment

The genome assemblies in our dataset exhibited substantial variations in size, ranging from 30 Mb of *Agaricus bisporus* to 16 Gb of *Allium sativum* (Supplementary Fig. S2a). Similarly, the assembly level demonstrated considerable variation from a chromosome-level assembly in *Citrus limon* with 9 chromosomes to a highly fragmented assembly in *Secale cereale*, consisting of 905,722 contigs (Supplementary Fig. S2b). The percentage of GC content varied widely between the genomes, with a mean of 37.43% and a standard deviation of 3.6% (Supplementary Fig. S2c).

### Completeness assessment

**Fig. 3:**
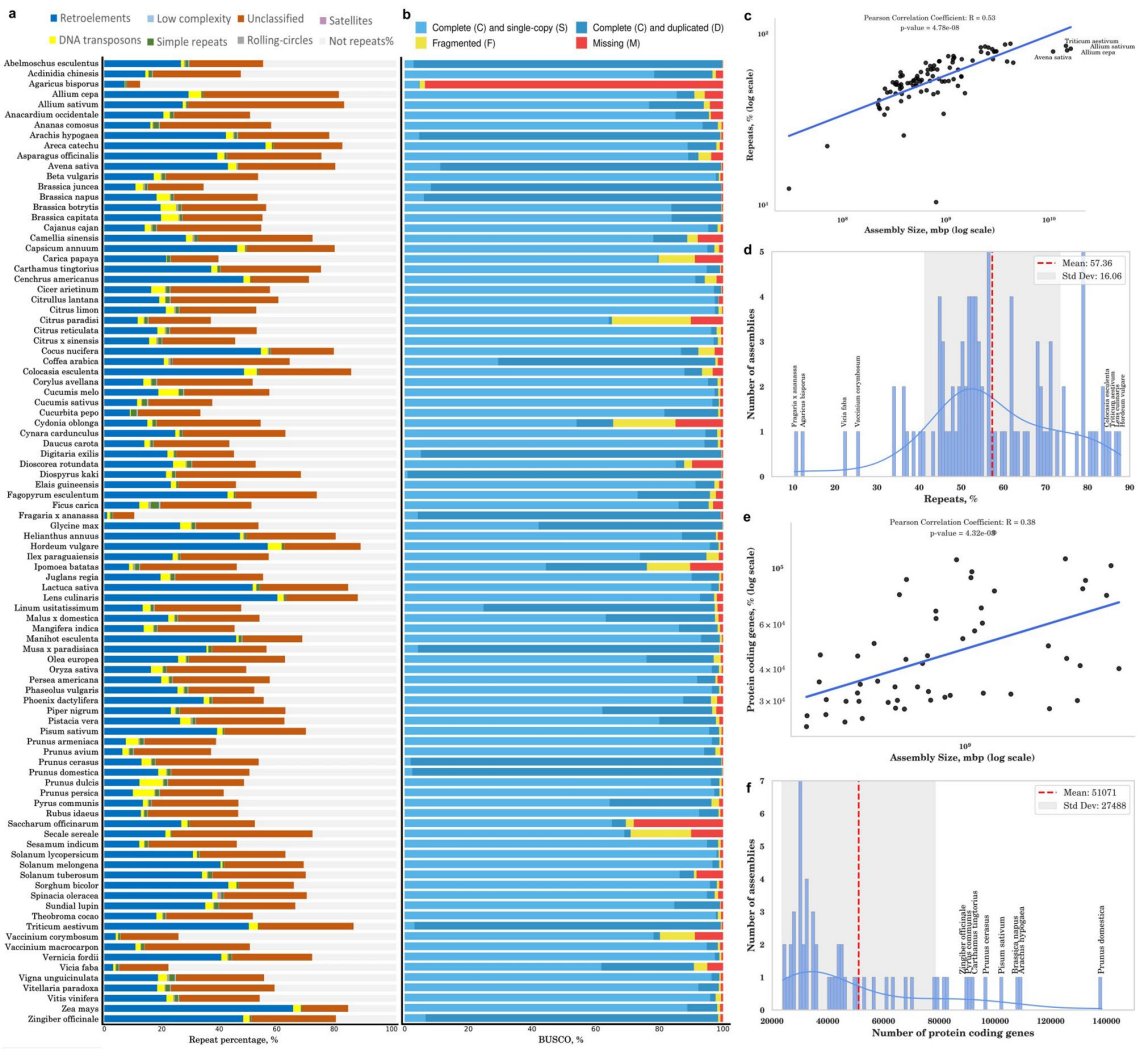
**(a)** Proportional representation of repeat families within the genome assemblies. **(b)** BUSCO assessment of plant assemblies showing relative completeness using the embryophyte lineage. Genes are grouped into five categories: complete (C), single-copy (S), duplicated (D), fragmented (F), or missing (M) BUSCO genes. **(c)** Correlation between assembly size and repeat content percentage. **(d)** Repeat content as a percentage of the total assembly size. **(e)** Correlation between assembly size and protein-coding gene count. **(f)** The number of protein-coding genes across 54 crop genomes

BUSCO, short for Benchmarking Universal Single-Copy Orthologs, is a widely used tool for evaluating the completeness of a genome assembly and its corresponding annotation [32]. It is designed to assess how well a genome assembly and its annotation present a particular lineage. This assessment relies on a predefined set of single-copy orthologous genes that are expected to be universally present in most genomes of that lineage. Although the set of genes, referred to as ‘BUSCO genes’, constitutes less than 5% of the entire gene pool of a particular genome, this method is particularly valuable for newly sequenced and for comparing the completeness of different genome assemblies or annotations. A quantitative assessment of the completeness in terms of the expected BUSCO gene content of a genome assembly demonstrated that, in general, the majority of crop genomes cover at least 90% of the BUSCO genes from the embryophyte lineage (Fig. 3b). A notable exception is constituted by *Agaricus bisporus*, a fungus that lacks a significant portion of genes specific to the embryophyte lineage of land plants. *Saccharum officinarum* (sugarcane) showed a lower completeness of only 70% for the embryophyte BUSCO genes. Such low completeness of the assembly may be attributed to the inherent complexities associated with the *Saccharum officinarum* genome, characterized by high levels of polyploidy, aneuploidy, and heterozygosity [61]. Additionally, ten crop genomes—*Abelmoschus esculentus*, *Brassica juncea*, *Avena sativa*, *Arachis hypogaea*, *Brassica napus*, *Digitaria exilis*, *Diospyros kaki*, *Fragaria x ananassa*, *Triticum aestivum*, and *Zingiber officinale—*exhibit a high percentage of duplicated BUSCO genes (over 90% duplication) (Supplementary Fig. S3). Possible reasons for this finding include the assembly of distinct haplotypes, a recent whole genome duplication event, or the presence of technical artifacts in the genome assembly process that require further investigation.

In our analysis, nine crop genome assemblies achieved ‘gold’ quality, and 36 assemblies achieved ‘reference’ quality as assessed by the LTR Assembly Index (LAI) score, while the majority (49 crop genomes) were classified as ‘draft’ quality (Supplementary Table 2). Notably, genomes in the draft category were predominantly sequenced without the aid of long-read sequencing technologies. Resequencing these genomes using advanced sequencing platforms and sophisticated assembly tools will significantly enhance the quality of these assemblies and benefit all subsequent downstream analyses.

### Analysis of repetitive elements

Crop genomes exhibited a high percentage of repetitive elements, averaging 57.36% across the genomes (Fig. 3d and Supplementary Table 3). Such high repeat content poses a major obstacle for assembling contigs into larger scaffolds. Overall, larger genome assemblies appeared to have higher repeat content, as indicated by a Pearson correlation coefficient of 0.53 (Fig. 3c). Among the families of repetitive elements, retroelements such as Long Interspersed Nuclear Elements (LINES), Short Interspersed Nuclear Elements (SINES), and Long Terminal Repeats (LTRs) are the most abundant, while DNA transposons constitute the second most prevalent family (Fig. 3a).

### The number of protein-coding genes

Based on the publicly available reference annotations for 54 crops, the number of protein-coding genes varies significantly, ranging from 23,802 in *Cucumis sativus* with an assembly size of 226 Mb to a maximum of 138,361 in *Prunus domestica* (Fig. 3f). Additionally, a high number of protein-coding genes were identified in the genomes of *Brassica napus, Pisum sativum var. sativum, Carthamus tingtorius, Pyrus communis*, and *Zingiber officinale*. Larger genomes tended to have more protein-coding genes (R = + 0.38) (Fig. 3e). However, it is important to note that genome size and the number of protein-coding genes do not always correlate with organismal complexity—a phenomenon referred to as the C-value/G-value enigma [62]. Furthermore, the observed positive correlation (R = + 0.61) between the proportion of duplicated BUSCO genes and the overall protein-coding gene count (Supplementary Fig. S4) might indicate technical artifacts in the genome assembly, such as misassembly or misplacement of contigs and scaffolds, which could falsely increase the apparent gene number. Such artifacts may arise from challenges in distinguishing between true gene duplication events and sequencing errors, particularly in the regions containing highly repeat content.

### Benchmarking gene prediction tools

**Fig. 4:**
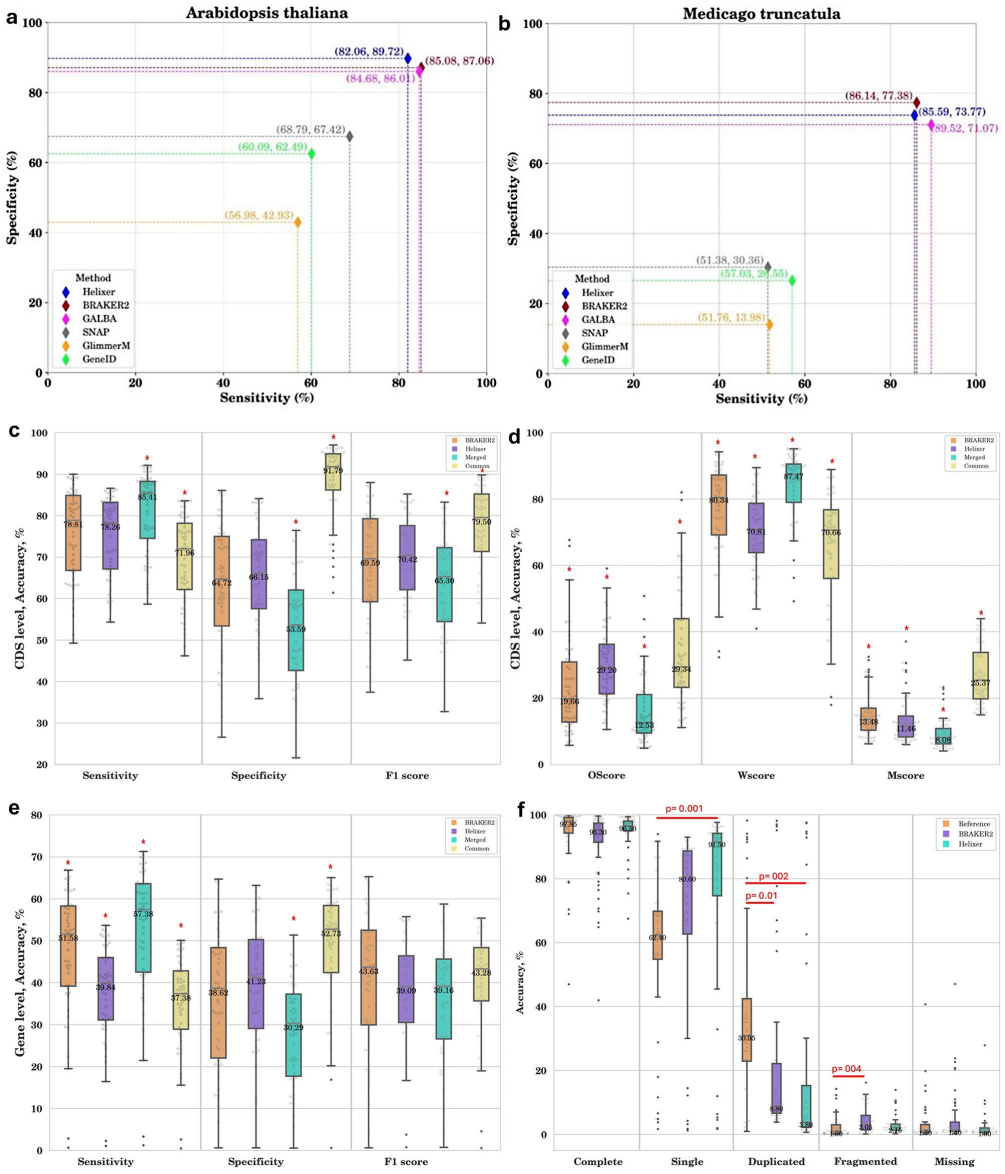
Sensitivity and specificity of gene prediction tools at the coding region (CDS) level on the *Arabidopsis thaliana***(a)** and *Medicago truncatula***(b)** genomes. **(c, d)** Evaluation of BRAKER2 and Helixer annotations at the coding region (CDS) level in 54 crops with available reference annotations. The figure displays a boxplot comparison across key performance metrics: Sensitivity, Specificity, F1 score, Overlapping exons score (OScore), Wrong exons score (WScore), and Missed exons score (MScore). The data are categorized by predictions unique to BRAKER2 (blue), unique to Helixer (green), those obtained by merging predictions from both tools (Merged, red), and common predictions by both tools (Common, yellow). **(e)** Evaluation of BRAKER2 and Helixer annotations at the gene level. **(f)** Evaluation of completeness scores for reference (orange), BRAKER2 (purple), and Helixer (green) annotations, highlighting the significant *p*-values

The benchmarking of the selected gene prediction tools was conducted against the well-curated reference annotations of the *Arabidopsis thaliana and Medicago truncatula* model plant genomes. These tools do not rely on species-specific transcriptomics or proteomics data. Comparative analysis revealed that BRAKER2, GALBA and Helixer exhibited superior performance in terms of sensitivity and specificity compared to the other tools assessed (Fig. 4a and 4b). While GALBA was specifically designed to work well with genomes that present challenges for BRAKER2—such as large genomes with abundant repeats and high GC content—it will not be included in this study and will be evaluated in future research.

BRAKER2 is an automated pipeline that uses protein evidence-supported self-training GeneMark-EP to generate a training gene set for the gene prediction tool AUGUSTUS, which employs generalized hidden Markov Models (GHMM) [41, 63–70]. On the other hand, Helixer adopts a different strategy, relying on a deep stacked Bidirectional Long Short-Term Memory (BLSTM) network to make predictions in the form of a base pair-wise classification, with subsequent generation of final gene models by HMMs. Given the comparable accuracies demonstrated by these two tools and their utilization of different methodologies for gene prediction, both BRAKER2 and Helixer were used in our assessment.

### Accuracy of BRAKER2 and Helixer at the CDS level

The accuracy of both BRAKER2 and Helixer was assessed at the CDS level against the reference annotations of 54 crops. Given the non-normal distribution of the data, as confirmed by the Shapiro-Wilk test, we opted for non-parametric tests. Specifically, we utilized the Kruskal-Wallis test to compare the distribution of each metric across the selected tools. Subsequently, we performed pairwise comparisons employing the Mann-Whitney U test to identify specific differences between the tools.

Our comparative analysis at the CDS level revealed no significant difference between BRAKER2 and Helixer in terms of sensitivity and specificity (*p* = 0.54 and 0.63, respectively). However, Helixer demonstrated superior ability to recognize exon-containing regions, as evidenced by its higher OScore and lower WScore compared to BRAKER2 (*p* = 0.0005). This suggests that while Helixer excels at identifying potential coding regions, it may not accurately predict the exact boundaries of them. This was further confirmed by the lower MScore, indicating that Helixer missed fewer coding regions than BRAKER2 (*p* = 0.04). Potential errors in annotating these regions in reference annotations could influence the assessment of gene prediction accuracy. A merged annotation approach, integrating outputs from both tools, notably enhanced sensitivity by 5–10%; however, this gain was offset by a decrease in specificity due to the inclusion of false positives from each method. An alternative strategy employing only the overlapping predictions from both tools significantly enhanced specificity by 30%, albeit at the cost of reducing sensitivity by 5–15%, as it excluded correct unique predictions from each individual tool (Fig. 4c and 4d).

### Accuracy of BRAKER2 and Helixer at the gene level

Our findings at the gene level indicate that, in terms of specificity, there was no significant difference between the tools (*p* = 0.42). However, BRAKER2 exhibited higher sensitivity, surpassing that of Helixer by approximately 10% (*p* = 3.8e-05). This suggests that BRAKER2 predicted proteins have fewer errors or frameshifts compared to Helixer. Combining the outputs of both tools resulted in a 7% boost in sensitivity, albeit at the cost of a proportional decrease in specificity. Furthermore, common genes predicted by both tools showed higher specificity but lower sensitivity (Fig. 4e).

### Completeness of predicted gene sets

We further assessed the quality of the reference annotations as well as of BRAKER2, and Helixer predictions using BUSCO assessment at the protein level for the embryophyte genes (Fig. 4f). Reference annotations demonstrated the highest completeness levels, with only five crops—*Camellia sinensis*, *Carica papaya*, *Cocos nucifera*, *Piper nigrum*, and *Saccharum officinarum*—exhibiting a poor completeness score below 80%. However, reference annotations had the highest rate of duplicated BUSCO genes. In comparison, BRAKER2 predictions had the poorest completeness scores and the highest rate of fragmented BUSCO genes. Helixer, on the other hand, excelled in predicting complete single copy BUSCO genes. All three annotations showed a very low percentage of missing BUSCO genes less than 2%. Notably, *Saccharum officinarum*’s annotations had the poorest quality, with only 47%, 42%, and 68% completeness reported for the reference, BRAKER2, and Helixer annotations, respectively. This observation aligns with the low completeness score of the *Saccharum officinarum* genome assembly, a factor likely attributed to the inherent complexities discussed earlier.

### Factors affecting gene prediction accuracy

**Fig. 5:**
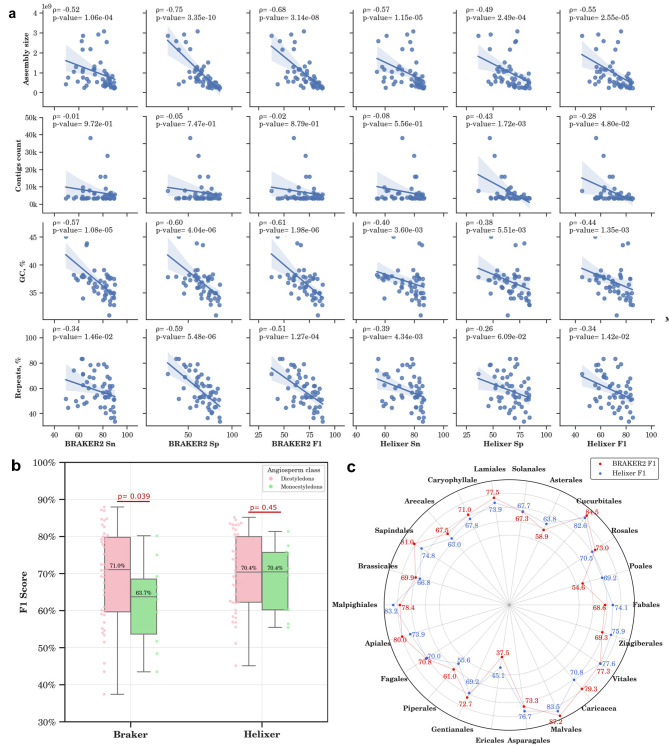
**(a)** Impact of genomic features on the accuracy of gene prediction. Each panel represents a scatter plot with a fitted linear regression line, showing the relationship between BRAKER2 and Helixer annotation metrics (Sensitivity - Sn, Specificity - Sp, and F1 Score) and key genome characteristics (Assembly Size, Contig Count, GC Content percentage, Repeat content). The Spearman correlation coefficient (R) and associated *p* values are displayed for each relationship, indicating the strength and significance of the correlations, with red asterisks highlighting the metric most impacted by each factor. **(b)** Comparison of BRAKER2 and Helixer in predicting genes within dicotyledons and monocotyledons classes, as indicated by F1 scores at the coding region (CDS) level. **(c)** Accuracy of BRAKER2 and Helixer predictions across different taxonomic plant orders, as demonstrated by average F1 scores at the coding region (CDS) level

To understand the factors influencing the performance of each tool, we examined the correlation between various metrics, including assembly size, the number of contigs, GC content percentage, and repeat content percentage, with the accuracy of both BRAKER2 and Helixer at the CDS level (Fig. 5a). Our findings indicate that the accuracy of both tools drops significantly for assemblies of larger size, especially impacting BRAKER’s specificity, which exhibits a Spearman correlation coefficient of -0.75 with the assembly size. This could be partly due to the reliance of BRAKER2 on protein alignment for guiding gene prediction, a method that might become less accurate as the complexity of the assembly increases. Moreover, the sensitivity of BRAKER2 was significantly inversely correlated with the GC content (ρ=-0.6). The specificity of Helixer is influenced by the number of contigs, with a correlation coefficient of -0.46, suggesting that its LSTM-based approach, while robust in handling long sequences, may struggle with fragmented genomes, as fragmentation can lead to incomplete or misassembled genes, challenging Helixer’s ability to accurately identify gene boundaries. At the same time, Helixer was more resistant to variations in GC content. The high content of repeats negatively affected both BRAKER2 and Helixer performance, with BRAKER2’s sensitivity being primarily affected (ρ=-0.59). These findings suggest that BRAKER2 may not be the optimal choice for projects involving large genomic assemblies with high GC content and high repeat content, as its ability to accurately identify specific gene sequences diminishes. In contrast, Helixer’s LSTM-based approach shows potential strengths in processing diverse genomic sequences but faces challenges with fragmented genomes. It is important to highlight that BRAKER2 can predict different protein isoforms arising from a single gene due to alternative splicing, whereas Helixer is limited to predicting a single protein isoform per gene. Moreover, BRAKER2 offers quantitative scores for its predicted features, facilitating the assessment of prediction confidence, unlike Helixer, which does not provide feature prediction scores.

We extended our analysis to explore the impact of angiosperm class on the accuracy of the two tools. This aspect is particularly crucial given the inherent genomic distinctions between monocotyledons and dicotyledons, such as the GC content of transcripts and the free energy of DNA promoters [71].

Our findings revealed that the accuracy of Helixer remained largely unaffected by the angiosperm class (Fig. 5b). However, when it came to BRAKER2 predictions on monocotyledons, a significant decrease in the F1 score, as much as 10%, was evident. This decrease in performance could potentially be attributed to BRAKER2’s reliance on protein hints from external databases. These databases are possibly skewed towards dicotyledons proteins, given the greater species diversity in dicotyledons compared to monocotyledons (75% and 22% of species diversity, respectively [72]). This significant difference in the number of dicotyledon species may have led to a more comprehensive and representative collection of dicotyledons protein data, thereby enhancing the accuracy of BRAKER2 for dicotyledons but not for monocotyledons.

### Gene prediction across different plant orders

We compared the accuracy of BRAKER2 and Helixer across various taxonomic plant orders (Fig. 5c). Notably, orders such as Malvales and Cucurbitales demonstrated high F1 scores for both BRAKER2 and Helixer, indicating robust gene prediction capabilities. In contrast, orders including Ericales, Asterales, and Poales exhibited lower accuracies with both tools. This may be attributed to various complexities inherent in the genomes of these taxa, which present challenges for accurate gene prediction. While the data suggests that BRAKER2 generally predicts coding regions with higher accuracy than Helixer in many of the represented orders, it is important to note that a statistical comparison was not feasible due to limited species representation in certain orders, with 11 orders containing fewer than three crops. A more thorough investigation into the genomic correlates of gene prediction accuracy within these taxa will be the subject of future study.

### Assessing the utility of predicted proteins for peptide identification in proteomics studies

**Fig. 6:**
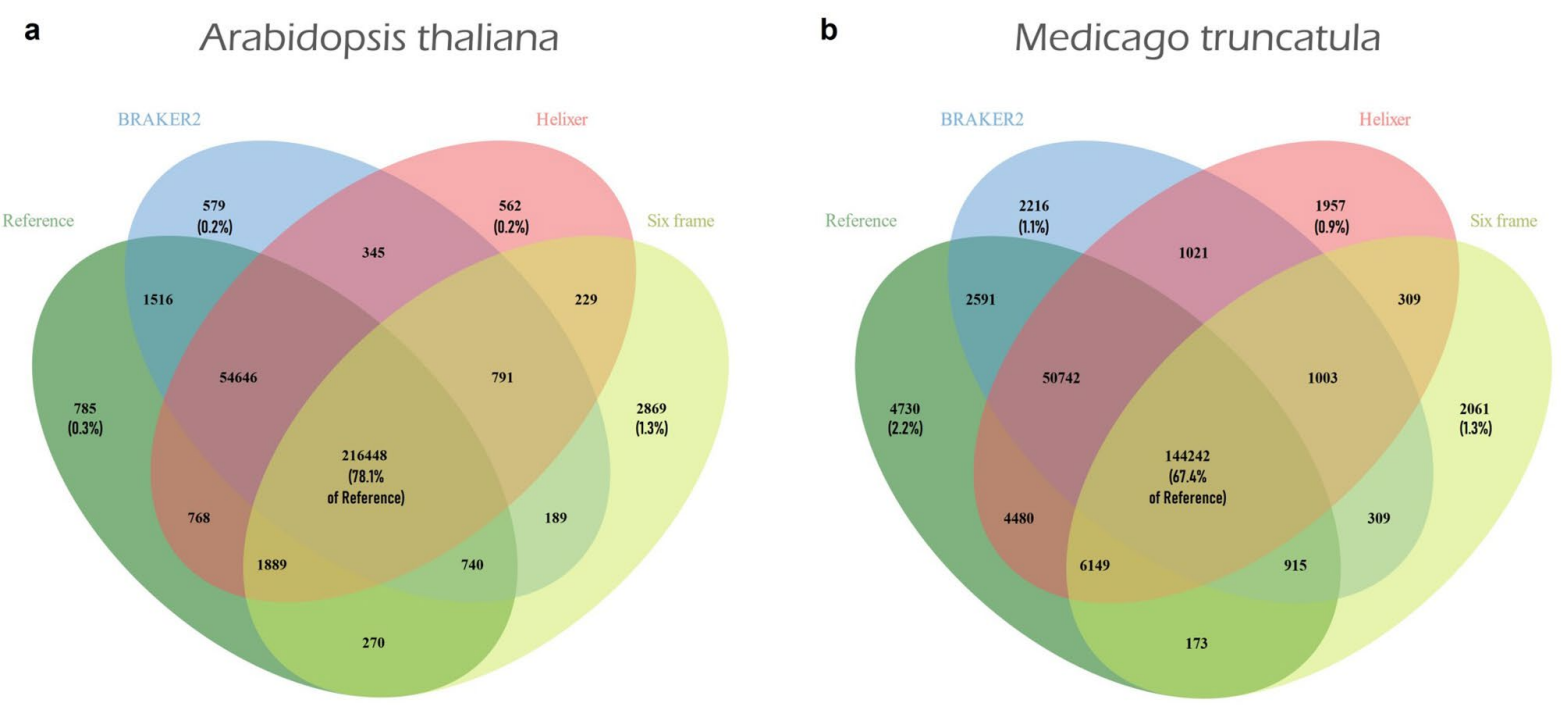
Overlaps between the peptide sets of *Arabidopsis thaliana* (**a**) and *Medicago truncatula* (**b**) identified from BRAKER2/Helixer predictions, a six-frame translation, and the reference annotation

We evaluated BRAKER2 and Helixer predictions for their suitability as a protein database for peptide identification in proteomics research using tandem mass spectrometry data. Fig. 6 illustrates the overlap between peptides identified using reference protein sequences and those predicted by BRAKER2 and Helixer, as well as the six-frame translation of the genome. Peptides identified by using the predicted proteins from BRAKER2 or Helixer exhibited a remarkable overlap with those identified using reference proteins. In *Arabidopsis thaliana*, both BRAKER2 and Helixer achieved a 99% overlap with the reference. In *Medicago truncatula*, BRAKER2 exhibited an overlap of 93% with the reference, while Helixer demonstrated a higher overlap of 96%. These results demonstrate that both BRAKER2-predicted proteins and Helixer-predicted proteins serve as effective search spaces for peptide identification in proteomics research. However, the discovery of peptides uniquely identified by one tool but not detected by the others underscores the inherent constraints and variability in gene prediction methodologies. Such unique, non-overlapping peptides may signal the presence of previously unidentified genes or gene isoforms, suggesting opportunities for enhancing the reference annotations with these novel peptides. The peptides identified with the six-frame translation of the genome displayed a lower overlap with those of the reference annotation, ranging from 67 to 77%. This can be attributed primarily to the lack of splice junction peptides in the six-frame translation. Additionally, the larger search space results in increased identification of false positive peptides, necessitating a stricter cutoff score at a 1% FDR, ultimately leading to the exclusion of a larger number of true positives as well. Notably, a small proportion (1–2%) of peptides identified through the six-frame translation exhibited no overlap with any other annotation search database. The fact that these peptides fall outside of annotated protein-coding regions indicates potentially incorrect exon boundaries, missed genes or coding exons, and can thus be used to indirectly assess the completeness of genome annotation. Furthermore, this underscores the importance of incorporating mass spectrometry data into genome annotation pipelines. Such integration holds promise for enhancing structural genome annotation and uncovering previously unannotated genes.

## Conclusions

In line with the goals of the “The Proteomes that Feed the World” project [27], which seeks to characterize the proteomic profiles of the top 100 crop plants essential for human nutrition using tandem mass spectrometry—a method reliant on high-quality genome annotations—this research systematically explored the genomes of these crops. Our study provides insights into the prevailing trends in genomic sequencing technologies and assembly methodologies. Notably, 40 of the 89 assembled plants lacked any publicly accessible reference annotation, underscoring the urgent need to find the best automated genome annotation strategy.

BRAKER2 and Helixer showed the best accuracy among the benchmarked genome annotation tools. Helixer stands out for its speed, independence from repeat masking, and applicability to large genomes. However, it exhibits lower performance on highly fragmented genomes assembled at the contig level. In contrast, BRAKER2 exhibits the capability to leverage extrinsic evidence into its annotations and shows superior sensitivity, especially at the gene level, with the ability to predict different protein isoforms per gene. However, BRAKER2 is more susceptible to challenges posed by high GC content and an abundance of repetitive elements, making it more suitable for small- to medium-sized genomes.

While our study has primarily explored specific gene prediction tools capable of ab initio predictions, either independently or with alignment hints derived from general protein databases, it is imperative to consider alternative and complementary approaches that could enhance gene prediction accuracy further. For instance, integrating species-specific RNA-seq data from various tissues and developmental stages will provide transcriptional evidence of predicted genes and can uncover new genes that were not identified through ab initio prediction methods. Furthermore, comparative genomics can aid in identifying conserved genes across related species, which is particularly beneficial for genomes that are less characterized. Additionally, the adoption of deep learning techniques, which have shown promise in tasks such as pattern recognition and predictive modeling, could be explored more thoroughly. Employing ensemble methods that combine multiple algorithms can also enhance prediction accuracy by leveraging their respective strengths and mitigating their weaknesses. Lastly, integrating proteomic data represents a significant opportunity to improve and refine genome annotation methodologies, thereby advancing the field of plant genomics.

## Electronic supplementary material

Below is the link to the electronic supplementary material.

Supplementary Material 1

Supplementary Material 2

Supplementary Material 3

Supplementary Material 4

Supplementary Material 5

## Data Availability

All assembly and annotation files were deposited in Zenodo under accession 10615294 (https://zenodo.org/records/10615294).

## Abbreviations

DNA: Deoxyribonucleic acid
RNA: Ribonucleic acid
CDS: coding sequence
LTR: Long Terminal Repeats
LAI: LTR Assembly Index
BUSCO: Benchmarking Universal Single-Copy Orthologs
HMM: Hidden Markov Model
TP: True positive
FP: False positive
TN: True negative
Sn: Sensitivity
Sp: Specificity
OScore: Overlapping exons score
WScore: Wrong exons score
MScore: Missed exons score
*p*-value: Probability value
MS: Mass spectrometry
FDR: False Discovery Rate
PSM: Peptide-Spectrum match
