# Correction: Exploring crop genomes: assembly features, gene prediction accuracy, and implications for proteomics studies

**DOI:** 10.1186/s12864-024-10796-z

**Published:** 2024-09-19

**Authors:** Qussai Abbas, Mathias Wilhelm, Bernhard Kuster, Brigitte Poppenberger, Dmitrij Frishman

**Affiliations:** 1 Chair of Bioinformatics, TUM School of Life Sciences, Technical University of Munich, Freising, Germany; 2 Computational Mass Spectrometry, TUM School of Life Sciences, Technical University of Munich, Freising, Germany; 3 Munich Data Science Institute, Technical University of Munich, Garching, Germany; 4 Chair of Proteomics and Bioanalytics, TUM School of Life Sciences, Technical University of Munich, Freising, Germany; 5 Biotechnology of Horticultural Crops, TUM School of Life Sciences, Technical University of Munich, Freising, Germany

**Correction: BMC Genomics 25, 619 (2024)**

10.1186/s12864-024-10521-w

Following publication of the original article, it was noticed that the sensitivity and specificity values of GALBA were reported incorrectly, leading to an error in Fig. 4, and 4b. The incorrect and correct versions of Fig. 4a and b. are given in this correction article.

**Incorrect figure panels**:

**Figure 1:**
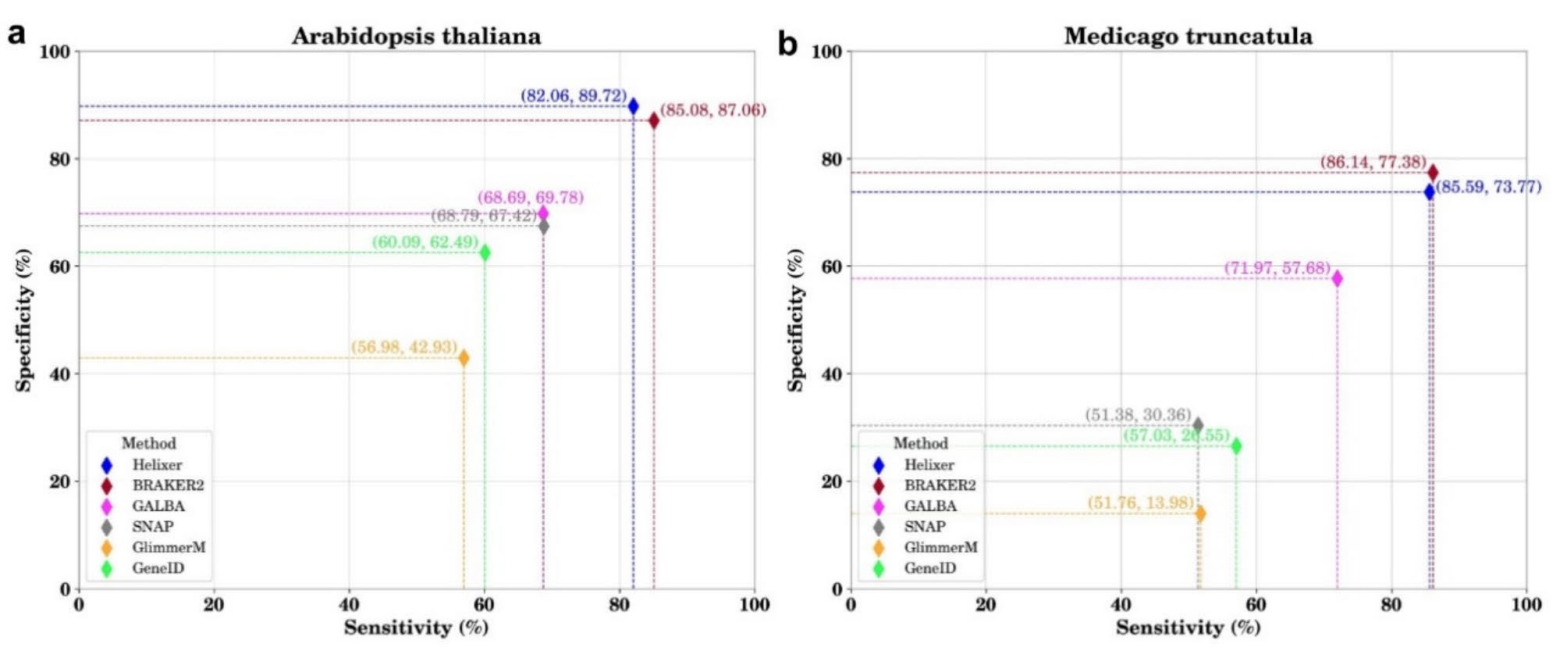

**Correct Fig. 4a and b**:

**Figure 2:**
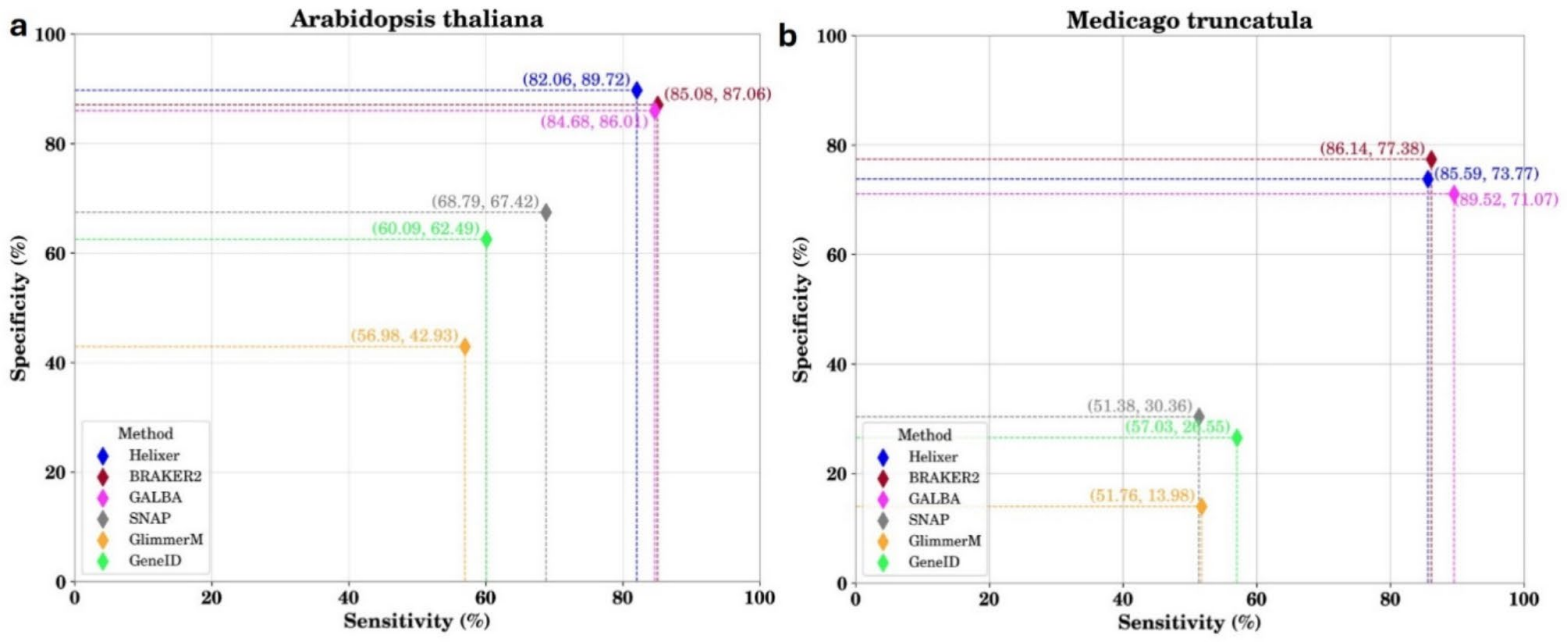

The following text in the ‘Benchmarking gene prediction tools’ relating to Fig. 4 has been corrected as a result of this correction.

**Original text**:

The benchmarking of the selected gene prediction tools was conducted against the well-curated reference annotations of the *Arabidopsis thaliana* and *Medicago truncatula* model plant genomes. These tools do not rely on species-specific transcriptomics or proteomics data. Comparative analysis revealed that BRAKER2, GALBA and Helixer exhibited superior performance in terms of sensitivity and specificity compared to the other tools assessed (Fig. 4a and b).

**Corrected text**:

The benchmarking of the selected gene prediction tools was conducted against the well-curated reference annotations of the Arabidopsis thaliana and Medicago truncatula model plant genomes. These tools do not rely on species-specific transcriptomics or proteomics data. Comparative analysis revealed that BRAKER2, GALBA and Helixer exhibited superior performance in terms of sensitivity and specificity compared to the other tools assessed (Fig. 4a and b). While GALBA was specifically designed to work well with genomes that present challenges for BRAKER2—such as large genomes with abundant repeats and high GC content—it will not be included in this study and will be evaluated in future research.

The original article has been updated.

