# Supplementary material for: Exploring crop genomes: assembly features, gene prediction accuracy, and implications for proteomics studies
Source: BMC Genomics. 2024 Jun 19;25:619. doi: 10.1186/s12864-024-10521-w (PMC11186247; doi:10.1186/s12864-024-10521-w)
Supplement: Supplementary file 5 — Supplementary Material 5 [file 12864_2024_10521_MOESM5_ESM.docx]

**Supplementary File 1**

**Exploring crop genomes: assembly features, gene prediction accuracy, and implications for proteomics ‎studies**

*Qussai Abbas, Mathias Wilhelm, Bernhard Kuster, Brigitte Poppenberger, and Dmitrij Frishman^§^*

*^$^* ‎Corresponding author: dimitri.frischmann@tum.de


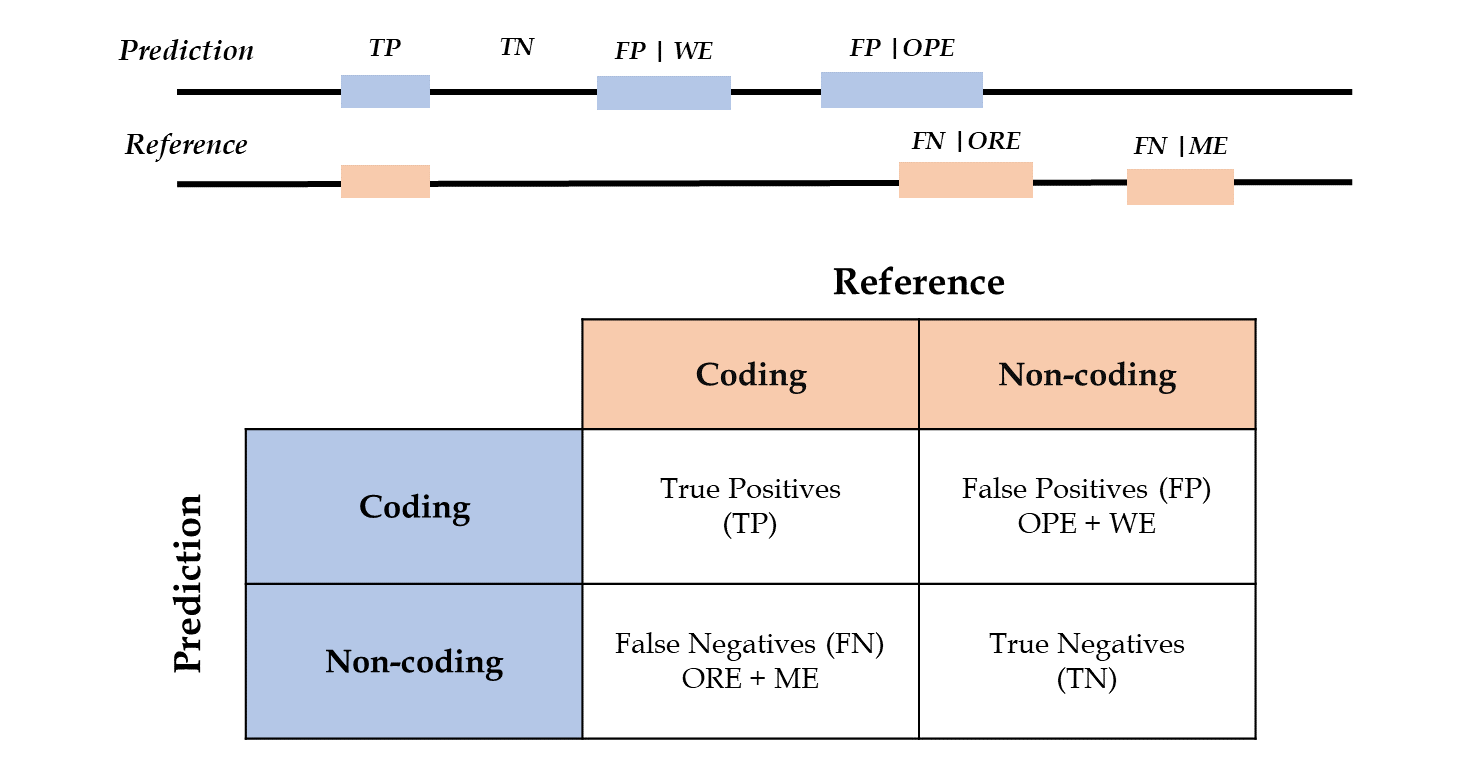


**Fig. S1.** Assessment of the accuracy of gene prediction tools at the coding region (CDS) level. True positives (TP) are ‎predicted coding regions that precisely match the reference coding regions. True negatives (TN) are coding ‎regions correctly identified as non-coding regions. False positives (FP) are divided into overlapping predicted ‎exons (OPE), which partially match reference coding regions, and wrong exons (WE), which do not ‎correspond to any reference exon. False negatives (FN) consist of overlapping reference exons (ORE), which ‎are reference exons partially covered by predictions, and missing exons (ME), which are reference exons not ‎predicted at all.‎


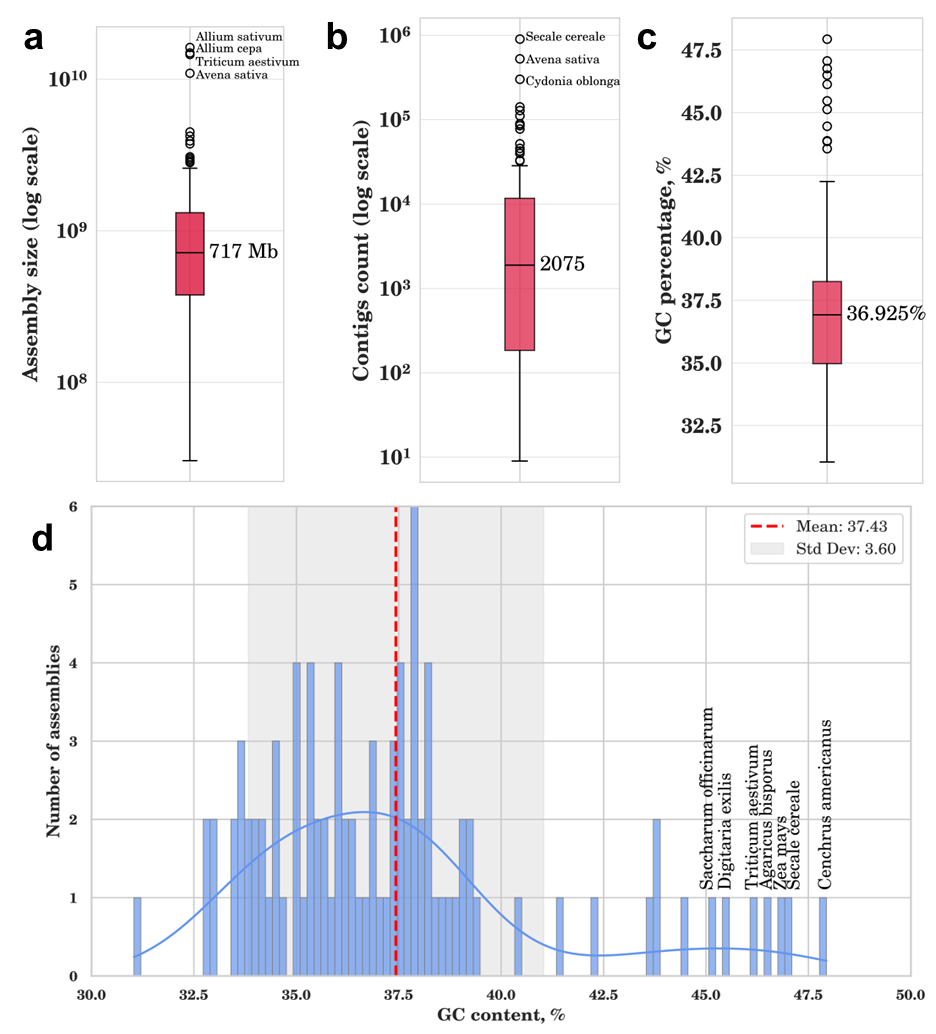


**Fig. S2. Genomes statistics (a)** Assembly size **(b)** contig count **(c)** GC content **(d)** Histogram distribution of GC content.


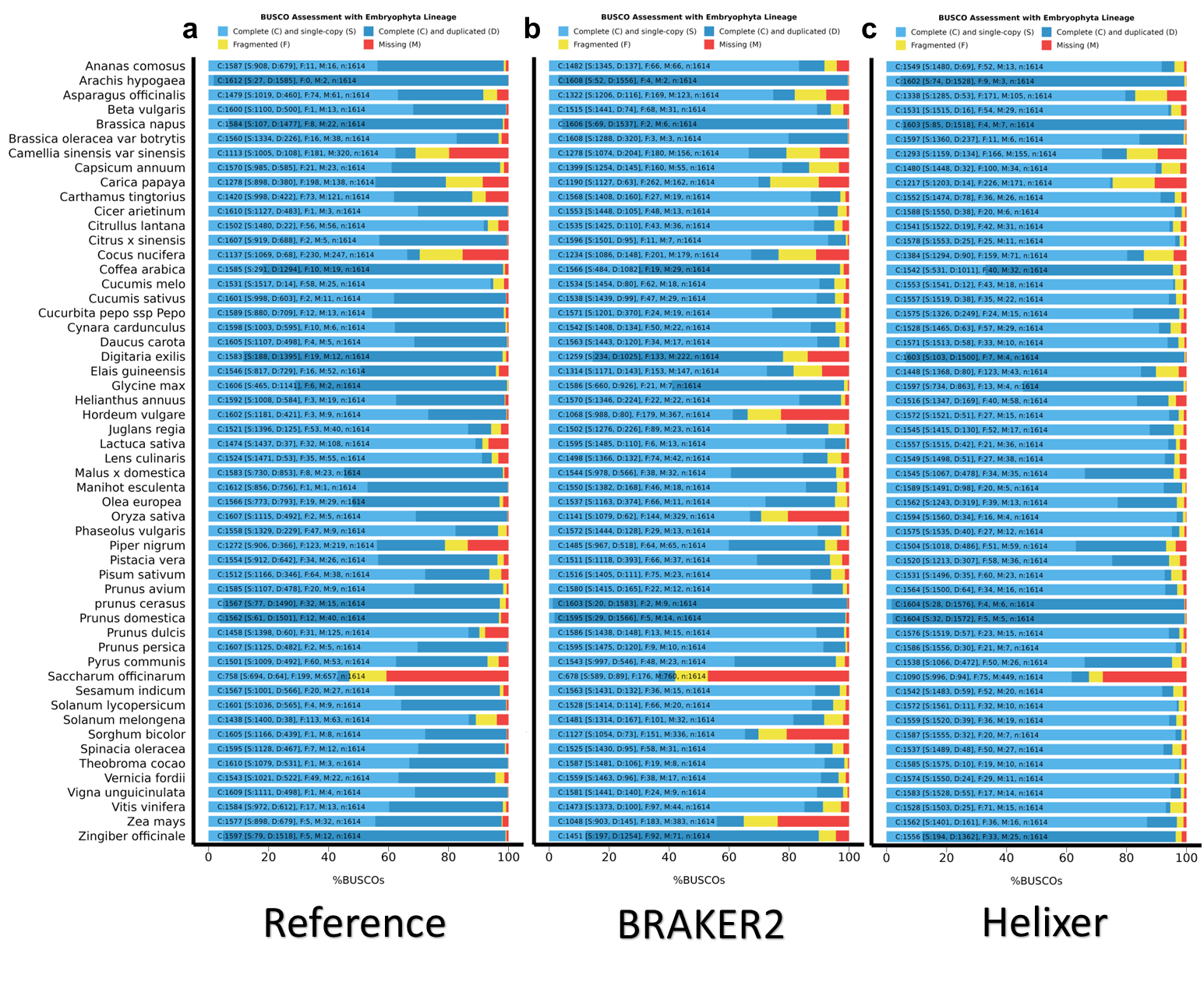


**Fig. S3. BUSCO assessment at the protein level of reference (a), BRAKER2 (b) and Helixer (c) annotations.**


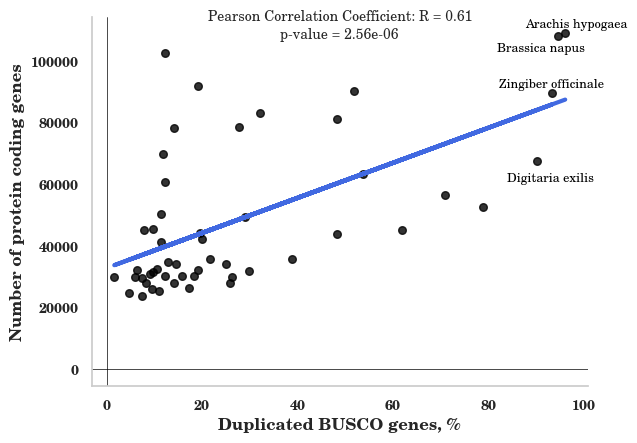


**Fig S4.** Correlation between number of protein coding genes and duplicated BUSCO genes.

**LAI index:**

LAI index was computed as follows:

gt suffixerator -db $genome -indexname $genome -tis -suf -lcp -des -ssp -sds -dna

gt ltrharvest -index $genome -minlenltr 100 -maxlenltr 7000 -mintsd 4 -maxtsd 6 -motif TGCA -motifmis 1 -similar 85 -vic 10 -seed 20 -seqids yes > $genome.harvest.scn

LTR_FINDER_parallel -seq $genome -threads 64 -harvest_out -size 1000000 -time 300

cat $genome.harvest.scn $genome.finder.combine.scn > $genome.rawLTR.scn

LTR_retriever -genome $genome -inharvest $genome.rawLTR.scn -threads 26 -Nscreen

LAI -genome $genome -intact $genome.pass.list -all $genome.out

**Gene prediction:**

Genome annotation tools were executed with all parameters set to default values. GlimmerM, and SNAP were run using the Genome Sequence Annotation Server (GenSAS) at [*https://www.gensas.org/*](https://www.gensas.org/)

For running BRAKER2, we used the OrthoDB Viridiplantae protein database downloaded from [*https://bioinf.uni-greifswald.de/bioinf/partitioned_odb11/Viridiplantae.fa.gz*](https://bioinf.uni-greifswald.de/bioinf/partitioned_odb11/Viridiplantae.fa.gz). We run BRAKER2 as follows:

perl braker.pl --species=$species --softmasking --cores 32 --workingdir=$Work_Dir --genome genome_masked.fa --prot_seq=OrthoDB_viridiplantae.fa

For running GALBA, we also used the OrthoDB Viridiplantae protein database downloaded from [*https://bioinf.uni-greifswald.de/bioinf/partitioned_odb11/Viridiplantae.fa.gz*](https://bioinf.uni-greifswald.de/bioinf/partitioned_odb11/Viridiplantae.fa.gz). We run GALBA as follows:

perl galba.pl --threads=32 --workingdir=$Work_Dir --species=$species --genome=genome_masked.fa --prot_seq=OrthoDB_viridiplantae.fa

For running Helixer we set the lineage parameter to land plants and we executed it with the following command:

python Helixer.py --lineage land_plant --fasta-path genome_masked.fa  --species $Species --gff-output-path $Work_Dir/$Species.helixer.gtf

GeneID:

param_file=species.param

geneid -P $param_file masked_genome.fa -3 > output.gtf
